# Observation of dynamic atom-atom correlation in liquid helium in real space

**DOI:** 10.1038/ncomms15294

**Published:** 2017-05-04

**Authors:** W. Dmowski, S. O. Diallo, K. Lokshin, G. Ehlers, G. Ferré, J. Boronat, T. Egami

**Affiliations:** 1 Shull Wollan Center—Joint-Institute for Neutron Sciences, Oak Ridge National Laboratory and University of Tennessee, Oak Ridge, Tennessee 37831, USA; 2 Department of Material Science and Engineering, University of Tennessee, Knoxville, Tennessee 37996, USA; 3 Oak Ridge National Laboratory, Oak Ridge, Tennessee 37831, USA; 4 Department de Física, Universitat Politécnica de Catalunya, Campus Nord B4-B5, E-08034 Barcelona, Spain; 5 Department of Physics and Astronomy, University of Tennessee, Knoxville, Tennessee 37996, USA

## Abstract

Liquid ^4^He becomes superfluid and flows without resistance below temperature 2.17 K. Superfluidity has been a subject of intense studies and notable advances were made in elucidating the phenomenon by experiment and theory. Nevertheless, details of the microscopic state, including dynamic atom–atom correlations in the superfluid state, are not fully understood. Here using a technique of neutron dynamic pair-density function (DPDF) analysis we show that ^4^He atoms in the Bose–Einstein condensate have environment significantly different from uncondensed atoms, with the interatomic distance larger than the average by about 10%, whereas the average structure changes little through the superfluid transition. DPDF peak not seen in the snap-shot pair-density function is found at 2.3 Å, and is interpreted in terms of atomic tunnelling. The real space picture of dynamic atom–atom correlations presented here reveal characteristics of atomic dynamics not recognized so far, compelling yet another look at the phenomenon.

## Abstract (web summary)

Liquid helium can be treated as an ideal gas or a condensed liquid and displays intriguing features like Bose–Einstein condensation. Here the authors show that roton excitation reveals information on real space dynamic atom-atom correlations in superfluid helium, which could be used to benchmark models.

Since the discovery of superfluidity 80 years ago^1^,^2^ the physics of liquid ^4^He have been extensively studied. London^3^,^4^ first proposed that the superfluid behaviour arises from Bose–Einstein condensation (BEC) to the quantum-mechanical ground state. However, London's proposal was based upon the assumption that helium can be regarded as ideal gas, whereas liquid helium is far from ideal gas. Landau^5^ later proposed a celebrated model, known as the two-fluid model, based upon the dynamics of the roton mode but without ever referencing to BEC. Feynman^6^,^7^ and others tried to reconcile these two conflicting theories, while both BEC and roton were experimentally detected by neutron scattering^8^,^9^. Liquid helium is a condensed matter with density not much different from that of a crystalline solid^10^. Atoms interact through the dispersion force, and as a result only about 7% of helium condenses into the Bose–Einstein ground state^11^. Consequence of helium being a non-ideal system on the nature of BEC has been the subject of active studies up to today^12^,^13^,^14^,^15^,^16^,^17^. In ideal gas atoms have no spatial correlations. But in liquid they are strongly correlated, as described in terms of the same-time or snap-shot, pair density function (PDF), *g*(*r*), the probability of two atoms separated by distance *r* (ref. 18).

Interestingly the PDF is reported to change only slightly through the superfluid transition^19^,^20^, even though the excitation spectrum measured by inelastic neutron scattering (INS) changes markedly with temperature^12^,^13^,^14^,^15^,^21^. The PDF is obtained by the Fourier-transformation of the total structure function, *S*_total_(*Q*), where *Q* is momentum transfer in scattering (see Supplementary Note 1) and *S*_total_(*Q*) is related to the dynamic structure factor measured by INS, *S*(*Q*,*E*), where *E* (=*ħω*) is energy transfer, through the energy integration,


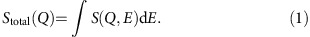


Therefore, the strong variation in *S*(*Q*,*E*) with temperature implies that the dynamic atomic correlation must be strongly dependent on temperature, even though they are almost totally masked when it is integrated over energy.

In this work the local atom–atom dynamic correlation in superfluid ^4^He was studied through the dynamic PDF obtained by the Fourier-transformation of *S*(*Q*,*E*). The results show that atoms involved in the BEC have environment different from the atoms in the normal state. Also a peak in the dynamic pair-density function (DPDF) at 2.3 Å was found, representing the coherent tunnelling action in the superfluid which could provide the mechanism of superfluidity in real space.

## Results

### Dynamic structure factor

**Figure 1:**
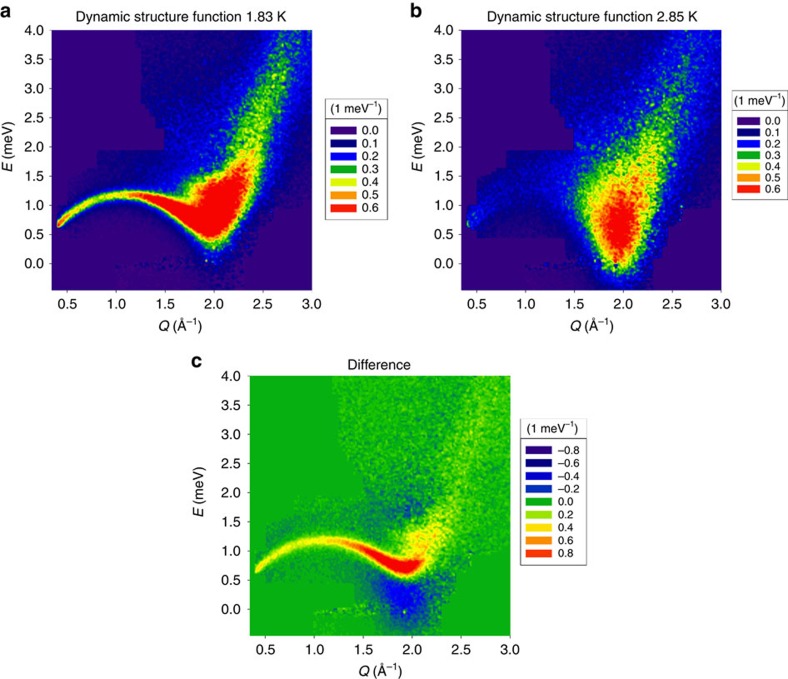
Dynamic structure factor. (**a**) *S*(*Q*, *E*) of ^4^He at *T*=1.83 K, (**b**) at *T*=2.85 K and (**c**) the difference, Δ*S*(*Q*, *E*)=*S*(*Q*, *E, T=*1.83 K)−*S*(*Q*, *E, T=*2.85 K), determined by inelastic pulsed neutron scattering. The unit is 1 meV^–1^.

This work is designed to observe directly such dynamic atom–atom correlations in liquid helium in real space. We measure *S*(*Q*,*E*) using INS, and obtain the information on real space dynamics through the Fourier-transformation of *S*(*Q*,*E*). In the past most of the data on the dynamics of ^4^He were obtained by the INS measurement at a reactor neutron source using a triple-axis spectrometer. In such a measurement only one neutron detector is used, allowing *S*(*Q*,*E*) to be measured at just one set of *Q* and *E* at a time. As a consequence it has been extremely time-consuming and challenging to determine *S*(*Q*,*E*) as a function of both *Q* and *E*. In contrast INS spectrometers with a pulsed neutron source have a large array of position sensitive detectors in the time-of-flight analysis mode, and can produce the two-dimensional (2D) data of *S*(*Q*,*E*) simultaneously over wide ranges of *Q* and *E* in a reasonable measurement time. We determined *S*(*Q*,*E*) for liquid ^4^He at selected temperatures (*T*=1.83, 1.93, 2.04, 2.35 and 2.85 K) at the saturated vapour pressure (SVP) using the cold neutron chopper spectrometer (CNCS) of the Spallation Neutron Source (SNS) at Oak Ridge National Laboratory using three different incident energies as described in Methods section. The data were corrected for absorption and background, mostly due to the sample holder, and were translated into *S*(*Q*,*E*). The three data sets of *S*(*Q*,*E*) with different incident energies were merged into one master *S*(*Q*,*E*), using as much as possible the data with the lower incident energies which had higher *Q* and *E* resolution. Figure 1 shows *S*(*Q*,*E*) of liquid ^4^He at *T*=1.83 K (in the superfluid state) and 2.85 K (normal state), and the difference between the two data sets, Δ*S*(*Q*,*E*)=*S*(*Q*,*E, T=*1.83 K)−*S*(*Q*,*E, T=*2.85 K). The sharpening of the roton excitations around *Q*_R_=1.98 Å^−1^ and *E*_R_=0.74 meV and the maxon excitations around *Q*_M_=1.1 Å^−1^ and *E*_M_=1.2 meV in the superfluid state is clearly seen, as observed before^21^.

### Snap-shot PDF

We calculated *S*_total_(*Q*) by equation (1) (integrated from −1 to 27 meV), and Fourier-transformed it to obtain the total (snap-shot) PDF, *ρ*_0_*g*_total_(*r*), where *ρ*_0_ is the atomic number density, as shown in Supplementary Fig. 1. According to (ref. 19) the height of the first peak of the PDF is slightly lower, by about 2%, in the superfluid state, and this was explained as a consequence of atomic delocalization in the superfluid. This interpretation was later questioned theoretically^22^. As discussed in Supplementary Note 1, in (ref. 19) the energy integration in equation (1) was not done at constant *Q*, because in their set-up of two-axis diffractometer *Q* varies with *E*. On the other hand we integrated correctly at constant *Q*, because we have a set of 2D data of *S*(*Q*,*E*). Our results do not support the conclusion in (ref. 19), and the peak height is nearly independent of temperature, consistent with the recent quantum-mechanical path integral Monte-Carlo (PIMC) simulation^23^. However, because of significant uncertainty in the result we can only raise a question about the validity of the widely cited conclusion in (ref. 19).

### Dynamic PDF

**Figure 2:**
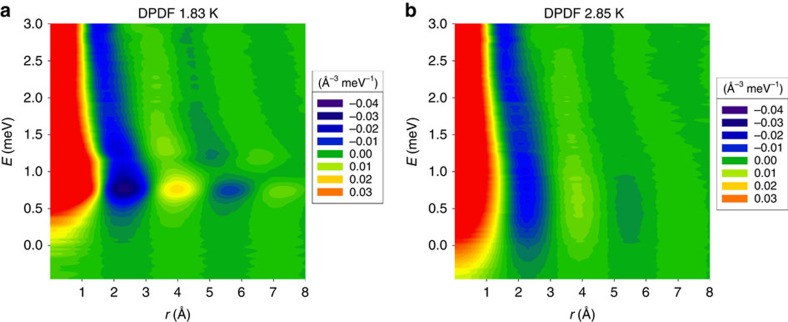
Dynamic pair-density function. (**a**) DPDF, *ρ*_0_[*g*(*r*, *E*)−1], of ^4^He at *T*=1.83 K and (**b**) at *T*=2.85 K. The unit is Å^−3^ meV^–1^. Zero corresponds to *g*(*r*, *E*)=1.

Whereas the total PDF shows small or no change with temperature, the details of dynamics are much more strongly dependent on temperature and exhibit significant variation through *T*_λ_. It is well known that the energy width of the phonon-roton excitation in *S*(*Q*,*E*) becomes drastically reduced below *T*_λ_, and becomes resolution limited^12^,^13^,^14^,^15^,^21^. However, the changes have not been interpreted in terms of local dynamics. In order to observe the local atomic dynamics we Fourier-transformed *S*(*Q*,*E*) into the DPDF, *ρ*_0_*g*(*r*,*E*) (refs 24, 25, 26), as discussed in Methods section and in Supplementary Note 2. The results are shown in Fig. 2 for *T*=1.83 K (below *T*_λ_) and 2.85 K (above *T*_λ_) as *ρ*_0_[*g*(*r*,*E*)−1]. Thus zero in Fig. 2 means *g*(*r*,*E*)=1. The strong intensities near *r*=0 are due to self-correlations. The bright spot around *r*=4 Å and *E*=0.7 meV is due to rotons. In addition, it is interesting to note that the intensity contours in DPDF are continuous at 2.85 K, but in the DPDF at 1.83 K discontinuity develops around 1 meV, indicating that the nature of roton (below 1 meV) excitations may be distinct from that of the excitations above 1 meV.

**Figure 3:**
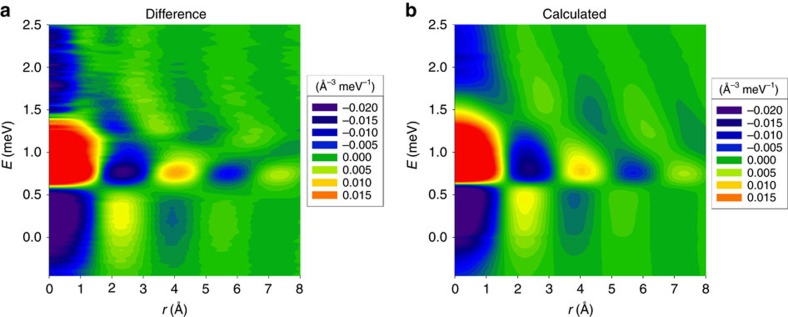
The difference in the DPDFs. (**a**) *ρ*_0_Δ*g*(*r*, *E*)=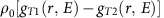, for the experimental data for *T*1=1.83 K and *T*2=2.85 K, (**b**) for the quantum Monte-Carlo (PIMC) simulation for *T*1=1.2 K and *T*2=2.5 K.

To illustrate the changes through *T*_λ_, *ρ*_0_Δ*g*(*r*,*E*), the difference in *ρ*_0_*g*(*r*,*E*) between *T*=1.83 K and 2.85 K, the superfluid state minus the normal state, is shown in Fig. 3a. The emergence of the peak related to sharpening of the roton excitations through *T*_λ_ is clearly seen in the energy range from 0.6 to 1 meV. In addition to the spot at 4 Å, a weaker spot at 7.5 Å is seen, both in *ρ*_0_*g*(*r*,*E*) at 1.83 K and in *ρ*_0_Δ*g*(*r*,*E*). The DPDF is most sensitive to local phonons and phonons near the saddle point of dispersion, whereas fast-dispersing phonons do not contribute much^25^. Thus rotons are most clearly seen in the DPDF, because they are close to the minimum of the dispersion *ω*_Q_, where d*ω*_Q_/d*Q*=0. In addition Fig. 3a shows that there is significant intensity centred around *r*_t_=2.3 Å and *E*_t_=0.4 meV. This peak was clearly seen even when the DPDF was derived only from the data with the incident energy of 3.5 meV or only from those with 15 meV (see Supplementary Note 5). Therefore it is highly unlikely that this peak is an artifact such as termination error. We compared the results with the DPDF obtained for the results of the PIMC calculation^23^. As shown in Fig. 3b the difference DPDF for the PIMC calculation is in very good agreement with the experimental one, with the roton peak at 4 Å and the peak at 2.3 Å clearly seen.

**Figure 4:**
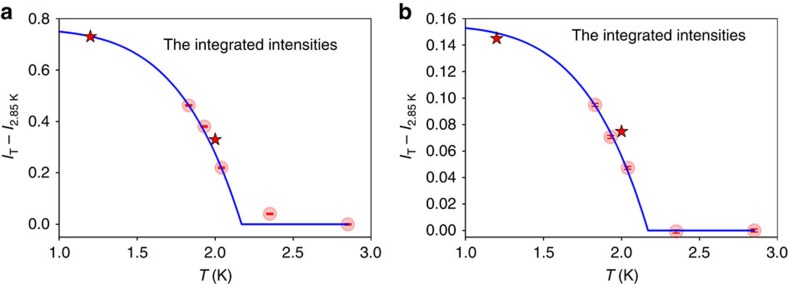
The integrated intensities. *I*(*T*) of the difference DPDF, Δ*g*(*r*, *E*), between the DPDF at temperature *T* and that at high temperature, 2.85 K for experiment and 2.5 K for simulation, (**a**) over the roton peak (*r*=3.4–4.6 Å, *E*=0.65–0.99 meV) and (**b**) the anankeon peak (*r*=2.0–2.6 Å, *E*=0.15–0.45 meV). Circles for experimental results and stars for PIMC simulation results. The solid lines are for the expected temperature dependence of the order parameter, equation (2), with *I*(0)=0.76 for the roton peak and 0.16 for the tunnelling peak. The vertical bars in circles indicate statistical error. The errors for the PIMC results are smaller than the symbol.

In Fig. 4 we show by circles the integrated intensity, *I*(*T*), of the difference between the DPDF at various temperatures and that at 2.85 K, (a) over the roton peak (*r*=3.4–4.6 Å, *E*=0.65–0.99 meV) and (b) for the peak at 2.3 Å (integrated for *r*=2.0–2.6 Å, *E*=0.15–0.45 meV). *I*(*T*) is shown in the unit of the coordination number, *N*_C_, which is 10.8 at 1.83 K and 10.6 at 2.85 K. *N*_*C*_ is defined by integrating 4*πr*^2^*ρ*_0_*g*(*r*) over the first peak in the PDF (Supplementary Fig. 1) up to the first minimum at 5.1 Å. It is seen that *I*(*T*) follows the temperature dependence of the BEC order parameter,


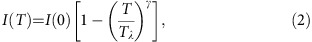


with *γ*=5.5 (ref. 11). We found *I* (0)=0.76±0.02 for the roton peak and *I* (0)=0.16±0.02 for the peak at 2.3 Å, shown by solid lines in Fig. 4. The value of *I*(*T*) for the roton peak at *T*=2.35 K is non-zero even though this temperature is above *T*_*λ*_. This must be due to local fluctuations naturally expected for the second-order transition. Figure 4 also shows the integrated peak intensity evaluated in the same way for the result of the PIMC calculation by stars^23^. They also follow the same temperature dependence. The fraction of atoms contributing to the roton peak, *I* (0)/*N*_C_, is 0.07±0.01, equal to the BEC fraction. The rest of the first peak in the total PDF (Supplementary Fig. 1) comes from the energy range other than the roton peaks. This result strongly suggests that the changes in *ρ*_0_*g*(*r*,*E*) in the energy range below 1 meV are directly due to atoms in the BE condensate.

### The peak at 4 Å

An interesting feature of the result is that the peak position of the roton excitation in *ρ*_0_Δ*g*(*r*,*E*), *r*_R,1_=4.0 Å, is significantly longer in distance than the peak of the total PDF, *r*_total_=3.65 Å, shown in Supplementary Fig. 1. The value of *r*_total_ is consistent with the earlier results^19^,^20^,^21^,^27^. The position of the second peak of the roton mode in *ρ*_0_Δ*g*(*r*,*E*), *r*_R,2_=7.5 Å, is also longer in *r* than that for the second peak of the total PDF at 6.7 Å. These increases in distance are far greater than any experimental error. The shifted positions for the peaks associated with sharpening of the roton are consistent with the observation that the volume of ^4^He liquid significantly expands below *T*_λ_ (ref. 10 (See Supplementary Note 3). This increased atomic distance gives, *r*_R,1_*Q*_R_=7.92, actually in agreement with the empirical formula, *rQ*=7.9–8.0 (ref. 28) (see Supplementary Note 4). Therefore it is the peak position of the total PDF, *r*_total_=3.65 Å (*r*_total_*Q*_R_=7.23), which is anomalous. In our view this is because BEC atoms are in the *q*=0 ground state, so the interatomic distances are close to the minimum of the van der Waals potential. On the other hand the distribution of atomic distances among uncondensed atoms is wide, as approximately given by the first peak of *g*_total_(*r*). But the first peak is strongly asymmetric in shape due to the repulsive part of the potential, so that the peak position underestimates the average. The van der Waals potential is very unharmonic, resembling the hard-core potential which is known to yield a low value of *rQ* (ref. 28).

### The peak at 2.3 Å

The peak at 2.3 Å was never seen in earlier studies. This distance is shorter than the hard-core radius for helium, 2.5 Å (ref. 29). Atoms so close to each other would experience very strong repulsive force, which is inconsistent with the low energy of this peak. On the other hand it should be noted that this distance is the furthest end of the one-body density matrix except for the long-range tail^11^,^12^. Therefore it is more likely that this peak is a part of the self-correlation,


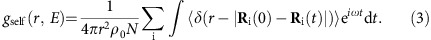


where **R**_i_(*t*) is the position of the *i*-th atom at time *t*, and ‘·····' represents thermal and quantum average. In Figs 2 and 3 the intensities close to *r*=0 are due to self-correlation because of self-motion such as vibration or diffusion. For instance, the strong intensity from 0.6 to 1.0 meV near *r*=0 is due to self-correlation for rotons. However, the peak at *r*_t_=2.3 Å is clearly separated from the intensity near *r*=0, and thus it cannot be due to vibrations, and is most likely due to tunnelling of atoms to cut or form atomic bonds (see Supplementary Note 5 for details). Such tunnelling occurs at all temperatures, but in the normal state the tunnelling action is random and the tunnelling distance is widely distributed. In the BEC state, however, it should become coherent and well-defined, because all BEC atoms are equivalent and take the same tunnelling action.

## Discussion

**Figure 5:**
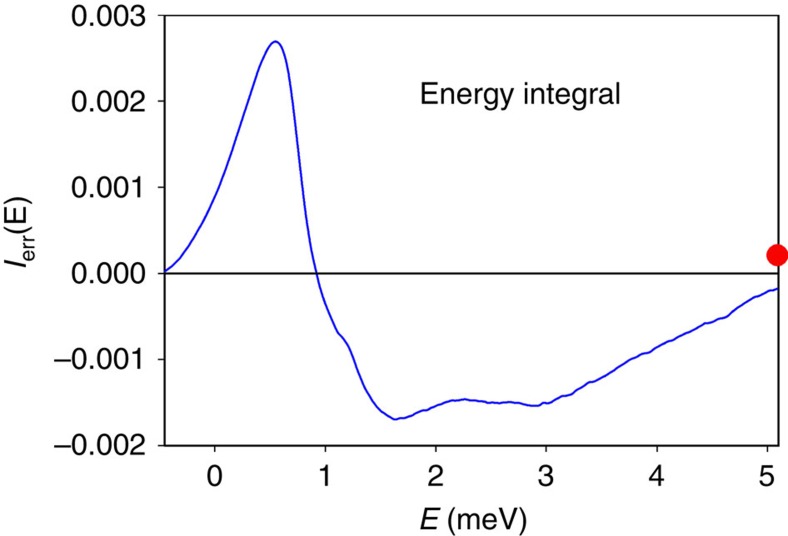
The integrated DPDF as a function of energy. The integral, *I*_err_(*E*) in equation (4), varies strongly with *E*, but converges to a small value at large *E*. The red dot indicates the value of *I*_err_(*E*) at the highest value of *E* in the present measurement, 27 meV.

Now the self-term of the same-time correlation function is a delta-function at *r*=0, and the peak at 2.3 Å should not appear in the total PDF if it is due to self-correlation. Indeed upon energy integration this peak up to *E*=0.6 meV is cancelled by the negative peak from 0.6 to 2 meV. To illustrate this cancellation we calculated the integrated DPDF, *I*_err_(*E*), defined by


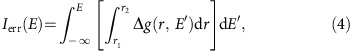


where *r*_1_=2.0 Å, *r*_2_=2.6 Å. *I*_err_(*E*) evaluates the contribution of the peak and valley in the DPDF at 2.3 Å to the total PDF. Ideally it should go to zero at *E*→∞. As shown in Fig. 5, it shows strong variation with *E*, but converges gradually to a small value at large *E*. Thus clearly there is a tendency of cancellation when integrated over energy. Although cancellation is imperfect because of the cutoff in energy integration and other errors, this tendency of cancellation strongly supports our conjecture that the peak at 2.3 Å in the DPDF originates from the self-correlation.

The low frequency excitations near *Q*_R_ are seen in almost all glasses and liquids, and are related to the so-called Boson peak in specific heat and Raman scattering^30^. *Q*_R_ is where there should be a Bragg peak in a corresponding crystal. In a crystal the phonon dispersion comes down to zero because *Q*_R_ is equivalent to *Q*=0 by the Umklapp operation. In liquids there is no Bragg peak in the absence of the translational symmetry, but instead there is a broad first peak in *S*_total_(*Q*). Consequently Umklapp is incomplete and these phonons near *Q*_R_ become diffuse and localized^31^.

In crystalline solids the elementary excitations of lattice vibration are phonons. In normal liquids, however, phonons are strongly scattered and marginalized because of dynamic structural disorder. Instead, the elementary excitations in the liquid were found to be local topological excitations in atomic connectivity network, the actions of cutting and forming atomic bonds^32^, named anankeons^33^. It is most likely that the excitations which contribute to the Boson peak around peak *Q*_R_ are anankeons. Indeed the possible connection between the Boson peak and topological fluctuation has been suggested^34^. Below *T*_λ_, however, phonons and rotons are well-defined and anankeons are suppressed. In particular the excitations in *S*(*Q*,*E*) near *Q*_R_ below the roton energy are suppressed by gap opening as shown in Fig. 1a,c. Because *r*_t_*Q*_R_∼1.5 *π* this peak contributes negatively to the DPDF, thus the suppression of the intensity in *S*(*Q*,*E*) near *Q*_*R*_ below the roton energy produces a positive effect in *ρ*_0_Δ*g*(*r*,*E*) around 2.3 Å, resulting in a relatively coherent peak. Because these excitations occur within the roton energy gap, they must represent tunnelling action as we argued above. Such coherent atomic displacements through collective tunnelling could be relevant to superfluid dynamics (see Supplementary Note 5). We plan to examine further the origin of this peak within the quantum Monte-Carlo simulation and further experiments.

In summary the atomic dynamics of superfluid ^4^He was studied through the DPDF method determined by inelastic pulsed neutron scattering. The results show that atoms involved in roton excitations below *T*_λ_ have longer interatomic distances than the average, and suggest that the atoms in the BE ground state have environment different from the average. A dynamic peak at 2.3 Å which was not recognized before was found, both in experiment and simulation. This peak is likely to represent the coherent tunnelling action in the superfluid which could facilitate the flow without viscosity. Our results present a local view of atomic dynamics of superfluid ^4^He, and call for renewed effort on theoretical interpretations of local atomic dynamics in the superfluid state.

## Methods

### Neutron scattering measurement

INS measurements were carried out using the CNCS of the SNS at Oak Ridge National Laboratory. INS measurements were made using three incident neutron energies of 3.5, 15 and 50 meV and at temperatures, 1.83, 1.93, 2.04, 2.35 and 2.85 K. The scattering data were collected for about 4 h for each incident energy and temperature. As the sample container a cylindrical aluminium container was used. The background including the empty container was measured at 2.5 K using three incident neutron energies. In addition vanadium standard was measured at 300 K to calibrate the detector efficiency. Subsequently, the container was filled with liquid ^4^He at SVP. The measured intensity, *I*(*Q*,*E*), was corrected for background and absorption. Then the data sets with different incident energies were scaled so that overlapping (*Q*,*E*) areas maintain the same integrated intensity, and then were merged to obtain the dynamic structure factor, *S*(*Q*,*E*), by utilizing the data sets with low incident energies, thus high *Q–E* resolution, as much as feasible. The data were put on an absolute scale by the condition *S*_total_(*Q*)→1 at high *Q*. The merged *S*(*Q*,*E*) covered the *Q–E* space up to 10 Å^−1^ and 30 meV.

### Dynamic pair-density function

The DPDF,


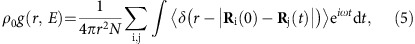


was obtained by the Fourier-transformation of *S*(*Q*,*E*) over *Q* (Supplementary Note 2). By integrating the DPDF over energy we obtain the same-time PDF,


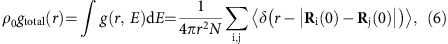


which is obtained by Fourier-transforming *S*_total_(*Q*). The idea of DPDF goes back to Carpenter and Pelizzari^35^, and was used in a different form for the analysis of dynamics in amorphous and crystalline solids^36^,^37^. The DPDF was successfully used in elucidating the dynamics of relaxor ferroelectrics^24^. We also Fourier-transformed *S*(*Q*,*E*) for each incident energy before merging, and confirmed that the salient features discussed here, such as the atomic distances for roton mode and the localized mode, are consistently seen with different resolutions (see Supplementary Note 5), except with the incident energy of 50 meV, where energy resolution was too low to discriminate these features.

### Data availability

The data acquired for this study (data for Figs 1, 2, 3, 4, 5 and Supplementary Figs 1–3) are included in the Supplementary Data files.

## Additional information

**How to cite this article:** Dmowski, W. *et al*. Observation of dynamic atom-atom correlation in liquid helium in real space. *Nat. Commun.* **8,** 15294 doi: 10.1038/ncomms15294 (2017).

**Publisher's note**: Springer Nature remains neutral with regard to jurisdictional claims in published maps and institutional affiliations.

## Supplementary Material

Supplementary Information Supplementary Figures, Supplementary Notes and Supplementary References.

Supplementary Data 1 Numerical listing of the data for Fig. 1 (a).

Supplementary Data 2 Numerical listing of the data for Fig. 1 (b).

Supplementary Data 3 Numerical listing of the data for Fig. 1 (c).

Supplementary Data 4 Numerical listing of the data for Fig. 2 (a).

Supplementary Data 5 Numerical listing of the data for Fig. 2 (b).

Supplementary Data 6 Numerical listing of the data for Fig. 3 (a).

Supplementary Data 7 Numerical listing of the data for Fig. 3 (b).

Supplementary Data 8 Numerical listing of the data for Fig. 4 (a) and (b).

Supplementary Data 9 Numerical listing of the data for Fig. 5.

Supplementary Data 10 Numerical listing of the data for Supplementary Fig. 1 Main

Supplementary Data 11 Numerical listing of the data for Supplementary Fig. 1 Inset.

Supplementary Data 12 Numerical listing of the data for Supplementary Fig. 2 (a).

Supplementary Data 13 Numerical listing of the data for Supplementary Fig. 2 (b).
