# Supplementary material for: Observation of dynamic atom-atom correlation in liquid helium in real space
Source: Nat Commun. 2017 May 4;8:15294. doi: 10.1038/ncomms15294 (PMC5418607; doi:10.1038/ncomms15294)
Supplement: Supplementary Information — Supplementary Figures, Supplementary Notes and Supplementary References. [file ncomms15294-s1.pdf]

**Supplementary Information for "Observation of dynamic atom-atom correlation in liquid helium in real space"**

## Supplementary Note 1: Total PDF

The total (snap-shot) PDF is obtained from the total structure function, $S_{\mathrm{total}}(Q)$, by

$$\rho(r)=\rho_0 g_{\mathrm{total}}(r)=\rho_0+\frac{1}{2\pi^2 r}\int\left[S_{\mathrm{total}}(Q)-1\right]\sin(Qr)QdQ\,, \qquad (1)$$

where $\rho_0$ is the average atomic number density (= 0.021 Å$^{-3}$). $S_{\mathrm{total}}(Q)$, or simply $S(Q)$, is usually determined by diffraction measurements without energy discrimination using a two-axis diffractometer. However, it is very challenging to determine accurately the atomic structure of liquid by x-ray or neutron diffraction for light elements such as helium. With x-rays it is difficult to measure $S(Q)$ up to high $Q$ because the elastic scattering intensity rapidly decreases with $Q$ and becomes overshadowed by the strong Compton scattering. For instance in Ref. 1 $S(Q)$ was determined only up to 4 Å$^{-1}$. For this reason the PDF of $^4$He which is currently considered to be the best was obtained by a neutron scattering experiment [2]. However, for neutron scattering challenges arise because of inelastic scattering, and in the regular two-axis diffraction measurement $S(Q)$ is distorted from the real one.

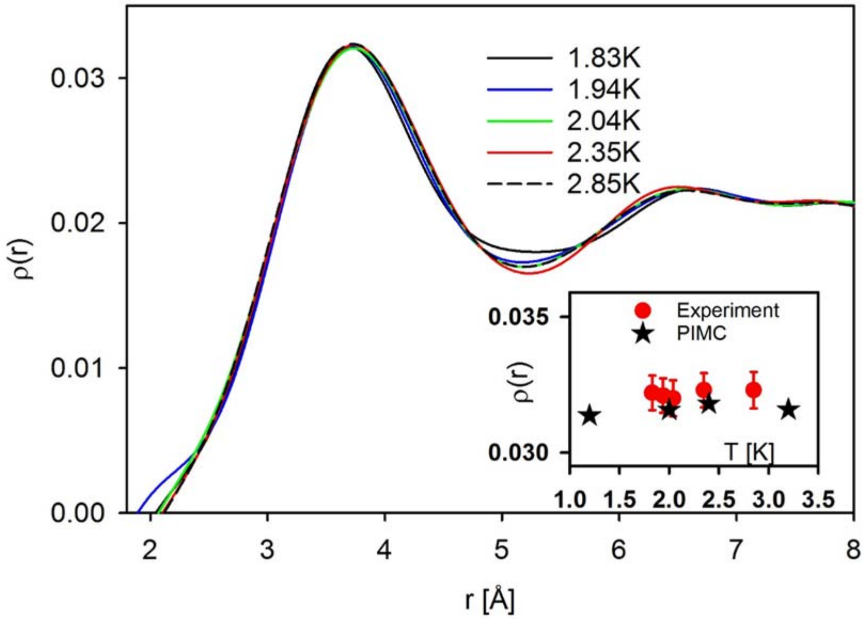


**Supplementary Figure 1 | Total PDF of liquid $^4$He at various temperatures.** The PDF is given in the unit of Å$^{-3}$. The inset shows the temperature dependence of the height of the first peak for experiment (red circles with error bars) and quantum Monte-Carlo simulation (black stars; error bars are smaller than the symbol). The error of the measured PDF (red error bar) was determined by the propagation of the statistical error [3].

When a detector is placed at an angle $2\theta$ from the incident beam the momentum exchange, $Q = |\boldsymbol{Q}| = |\boldsymbol{k}_\mathrm{i} - \boldsymbol{k}_\mathrm{f}|$, where $\boldsymbol{k}_\mathbf{f}$ and $\boldsymbol{k}_\mathbf{i}$ are the momenta of the scattered and incident neutrons, depends on the energy exchange $E$ ($= (\hbar^2/2m)\left[|\boldsymbol{k}_\mathrm{i}|^2 - |\boldsymbol{k}_\mathrm{f}|^2\right]$), where $m$ is the neutron mass. In a two-axis measurement without an energy analyzer a detector counts all neutrons with different energies, thus $S(Q, E)$ is integrated over energy. However, integration is not done at a constant $Q$, but $Q$ varies with $E$. Because the true value of $Q$ is not known the momentum exchange for elastic scattering, $Q_0 = 2k_\mathrm{i}\sin\theta$, is assigned as $Q$, in a rather gross approximation. Because of this effect the $S(Q)$ measured by a two-axis diffractometer is deviated from $S_\mathrm{total}(Q)$, and consequently $\rho_0 g(r)$ obtained this way is distorted from $\rho_0 g_\mathrm{total}(r)$. In Ref. 2 the authors attempted to correct this effect, but by considering only the neutron recoil by a free He atom. Therefore the distortion due to the actual dynamic correlations remained uncorrected.

In the present work we numerically integrate the measured $S(Q, E)$ over energy up to 27 meV as in eq. (1) to determine $S_\mathrm{total}(Q)$. Thus the energy integration is done at a constant $Q$. The total PDF obtained with supplementary eq. (1) is shown in Supplementary Figure 1. Because of the noise at high values of $Q$, $S(Q)$ was terminated at 5 Å$^{-1}$. The total PDF shown here is similar to earlier results. The inset shows the temperature dependence of the height of the first peak for the experiment and the quantum Monte-Carlo (PIMC) simulation. It is noted that the decrease in the peak height below $T_\lambda$ observed in Ref. 2 is not seen here. Because the energy integration is not done correctly in Ref. 2 as discussed above it is possible that the distortion introduced by the incorrect integration affected the result.

**Supplementary Note 2: Dynamic PDF method**

The dynamic PDF is obtained from $S(Q, E)$ by the Fourier-transformation from $Q$ to $r$ for three-dimensions [4-6]. However, in real experiments $S(Q, E)$ is determined only over a limited range of $Q$, up to $Q_\mathrm{max}(\omega)$, which depends on energy ($E = \hbar\omega$). Terminating the Fourier-transformation at a finite $Q$ results in error called termination error. In order to reduce significantly the termination effect we calculate,

$$\rho_0 g(r,\omega) = \frac{1}{2\pi^2 r}\int\left[S(Q,\omega) - S_\mathrm{asympt}(Q,\omega)\right]\sin(Qr)\,Q\,dQ\ , \qquad (2)$$

where $S_\mathrm{asympt}(Q, \omega)$ is the control function for the asymptote,

$$S_\mathrm{asympt}(Q,\omega) = A(\omega)\exp\left(-\frac{Q^2}{2\sigma(\omega)^2}\right). \qquad (3)$$

This function was chosen to be of the form of the self-part of $S(Q, E)$ of ideal gas, to which $S(Q, E)$ is known to approach asymptotically at large $Q$. Here $A(\omega)$ represents the energy dependence of the self-correlation function, and is determined so that $S(Q,\omega)$ - $S_\mathrm{asympt}(Q,\omega) = 0$ at $Q = Q_\mathrm{max}(\omega)$, and $\sigma(\omega)$ is the Debye-Waller factor, chosen in such a way that $S_\mathrm{asympt}(Q,\omega)$ fits smoothly to the measured data.

## Supplementary Note 3: Volume expansion

If the increase in the interatomic distance by BE condensation directly translates to volume expansion, the volume increase per BEC atom will be about 32%. This times the BEC fraction is 2.2%, consistent with the observed volume increase [7].

## Supplementary Note 4: Atomic distances

In supplementary eq. (2) $\sin(Qr)Qr$ has a maximum at $Qr = 7.98$, just above $(5/2)\pi = 7.85$. Therefore the peak in $g(r)$ at $r_p$ and the peak in $S(Q)$ at $Q_p$ are connected by $r_pQ_p \sim 8.0$ [8]. Judged from the value of $n_0(0)$ the DPDF peak at 4 Å and 0.7 meV seen in Fig. 3 is due to all the neighbors of the BEC atom. To explain this result there are two possible scenarios. One possibility is that this distance (4 Å) indeed represents the distance between BEC atoms. For this scenario to be valid, however, the BEC atoms have to be spatially segregated, which is highly unlikely. Another possibility is that the nearest neighbors of a BEC atom are mostly normal atoms but they behave like a BEC atom though the proximity effect. This scenario is much more

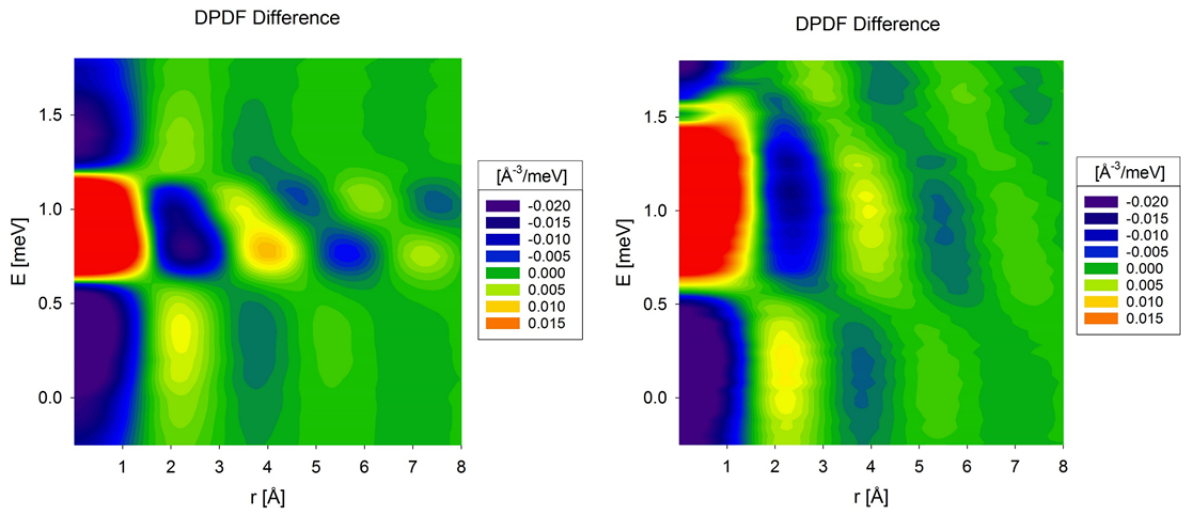


(a) (b)

**Supplementary Figure 2 | Change in the DPDF across the superfluid transition.** (**a**) $\rho_0\Delta g(r, E)$ for $T1 = 1.83$ K and $T2 = 2.85$ K similar to Fig. 3, but obtained only from the data with the incident neutron energy of 3.65 meV, and (**b**) with the incident neutron energy of 15 meV. The peaks at 4 Å and 0.8 meV and at 2.3 Å and 0.3 meV are seen in both data, in spite of the different $Q$ ranges for these sets of data.

likely.

## Supplementary Note 5: The peak at 2.3 Å in DPDF

As shown in Fig. 3 (a) and (b) there is a positive peak at 2.3 Å and 0.3 meV in $\rho_0\Delta g(r, E)$. Fig. 2 shows that at $T = 2.85$ K, $\rho(r = 2.3\text{ Å}, E = 0.3\text{ meV}) - \rho_0$ is about -0.02 Å$^{-3}$, meaning $\rho$(2.3 Å, 0.3

meV) ~ 0, whereas at $T$ = 1.83 K, $\rho$(2.3 Å, 0.3 meV) ~ 0.01 Å$^{-3}$. Therefore this peak is present only in the superfluid phase. The DPDF peaks at 4 Å and 0.7 meV, and at 2.3 Å and 0.3 meV, in Fig. 3 are clearly seen even when the DPDF was derived only from the data with the incident energy of 3.65 meV, or from those with 15 meV, with different $Q$ ranges for integration (Supplementary Figure 2). This peak was not resolved in the data with 50 meV incident energy, because of low $Q$ and $E$ resolution.

In the normal state above $T_\lambda$ the boson peak (around $Q_R$ and $E$ = 0 ~ 1 meV as seen in Fig. 1 (b)) broadly contributes to the DPDF intensity near the first peak and the self-correlation peak around $r$ = 0 of the DPDF due to random local displacements of atoms as shown in Fig. 2 (b). Below $T_\lambda$, however, the boson peak is replaced by the rotons. The loss of the boson peak produces a peak around $r_t$ = 2.3 Å. This is because the boson peak contributes negatively to the DPDF at $r_t$ = 2.3 Å; in supplementary eq. (2) below, $Q_R r_t$ ~ 3π/2, thus sin($Q_R r_t$) ~ -1. What this result indicates is that the topological excitations are random in the normal state, resulting in a Gaussian self-correlation peak. But they become more coherent in the superfluid, as represented by a well-defined peak at $r_t$. Because these excitations occur within the roton energy gap, they must represent tunneling action. It is possible that such atomic displacements through collective tunneling are relevant to superfluid dynamics.

One of the most common form of anakeons is the bond exchange (Supplementary Figure 3) [9-11]. The magnitude of the atomic displacement involved in such bond exchange in three-dimensions involving two tetrahedra is, $R_a = a\left(2\sqrt{2/3}-1\right)$, where $a$ is the atomic separation. For $a$ = 3.6 Å, $R_a$ = 2.3 Å, equal to $r_t$. In the normal state above $T_\lambda$ various patterns of anankeon excitations are possible, so $g_{self}(r, t)$ will be Gaussian with the width equal to $\sqrt{Dt}$ where $D$ is the diffusion constant. But in the superfluid it is likely that such well-defined coherent atomic displacements become dominant in the superfluid dynamics, and could provide the real space mechanism of flow in superfluid.

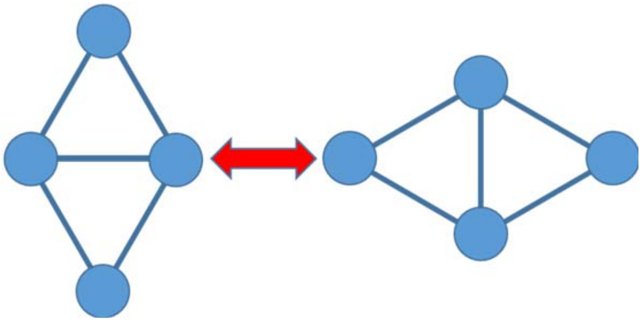

**Supplementary Figure 3 | Bond exchange model in two-dimensions [9-11].**

**References:**


1. Achter, F. K. and Meyer, L. X-ray scattering from liquid helium. *Phys. Rev.* **188,** 291-300 (1969).
2. Svensson, E. C., Sears, V. F., Woods, A. D. B. and Martel, P. Neutron-diffraction study of the static structure factor and pair correlations in liquid $^4$He. *Phys. Rev. B* **21,** 3538-3651 (1980).
3. Toby, B. H. and Egami, T. Accuracy of Pair Distribution Function Analysis Applied to Crystalline and Non-Crystalline Materials. *Acta Crystallogr. A* **48**, 336-346 (1992).
4. Dmowski, W., Vakhrushev, S. B., Jeong, I.-K., Hehlen, M. P., Trouw, F. and Egami, T. Local Lattice Dynamics and the Origin of the Relaxor Ferroelectric Behavior. *Phys. Rev. Lett.*, **100,** 137602 (2008).

5. Egami, T. and Billinge, S. J. L. *Underneath the Bragg Peaks: Structural Analysis of Complex Materials* (Pergamon Press, Elsevier Ltd., 2003); 2nd edition (2012).
6. Egami, T. and Dmowski, W. Dynamic pair-density function method for neutron and x-ray inelastic scattering. *Z. Krist.* **227,** 233-237 (2012).
7. Keesom, W. H. and Keesom, A. P. Thermodynamic diagrams of liquid helium. *Physica* **1,** 128-133 (1934).
8. Cargill, G. S., III. Structure of metallic alloy glasses. *Solid State Phys*. **30,** 227-320 (1975).
9. Suzuki, Y., Haimovic, J. and Egami, T. Bond-Orientational Anisotropy in Metallic Glasses Observed by X-Ray Diffraction. *Phys. Rev. B* **35**, 2162-2168 (1987).
10. Iwashita, T. and Egami, T. Atomic Mechanism of Flow in Simple Liquids under Shear. *Phys. Rev. Lett.*, **108,** 196001 (2012).
11. Egami, T. Elementary Excitation and Energy Landscape in Simple Liquids. *Mod. Phys. Lett. B*, **28,** 1430006 (2014).
